# Comparison of the gut microbiome and resistome in captive African and Asian elephants on the same diet

**DOI:** 10.3389/fvets.2023.986382

**Published:** 2023-02-16

**Authors:** Xin Feng, Rong Hua, Wanying Zhang, Yuhang Liu, Caiyu Luo, Tonghao Li, Xiaolin Chen, Hui Zhu, Youcong Wang, Yan Lu

**Affiliations:** ^1^School of Life Science and Engineering, Foshan University, Foshan, China; ^2^Beijing Key Laboratory of Captive Wildlife Technologies, Beijing Zoo, Beijing, China

**Keywords:** elephant, captivity, microbiome, resistome, diet

## Abstract

Elephants are endangered species and threatened with extinction. They are monogastric herbivorous, hindgut fermenters and their digestive strategy requires them to consume large amounts of low quality forage. The gut microbiome is important to their metabolism, immune regulation, and ecological adaptation. Our study investigated the structure and function of the gut microbiota as well as the antibiotic resistance genes (ARGs) in captive African and Asian elephants on the same diet. Results showed that captive African and Asian elephants had distinct gut bacterial composition. MetaStats analysis showed that the relative abundance of *Spirochaetes* (*FDR* = 0.00) and *Verrucomicrobia* (*FDR* = 0.01) at the phylum level as well as *Spirochaetaceae* (*FDR* = 0.01) and *Akkermansiaceae* (*FDR* = 0.02) at the family level varied between captive African and Asian elephants. Among the top ten functional subcategories at level 2 (57 seed pathway) of Kyoto Encyclopedia of Genes and Genomes (KEGG) database, the relative gene abundance of cellular community-prokaryotes, membrane transport, and carbohydrate metabolism in African elephants were significantly lower than those in Asian elephants (0.98 vs. 1.03%, *FDR* = 0.04; 1.25 vs. 1.43%, *FDR* = 0.03; 3.39 vs. 3.63%; *FDR* = 0.02). Among the top ten functional subcategories at level 2 (CAZy family) of CAZy database, MetaStats analysis showed that African elephants had higher relative gene abundance of Glycoside Hydrolases family 28 (GH 28) compared to Asian elephants (0.10 vs. 0.08%, *FDR* = 0.03). Regarding the antibiotic resistance genes carried by gut microbes, MetaStats analysis showed that African elephants had significantly higher relative abundance of *vanO* (*FDR* = 0.00), *tetQ* (*FDR* = 0.04), and *efrA* (*FDR* = 0.04) than Asian elephants encoding resistance for glycopeptide, tetracycline, and macrolide/rifamycin/fluoroquinolone antibiotic, respectively. In conclusion, captive African and Asian elephants on the same diet have distinct gut microbial communities. Our findings established the ground work for future research on improving gut health of captive elephants.

## Background

Elephants, including African elephant (*Loxodonta africana, Loxodonta cyclotis*) and Asian elephant (*Elephas maximus*), are considered endangered species and threatened with extinction (WWF, 2022). Captive elephants often experience health issues, such as gastrointestinal issues, low reproductive rate, high body condition, and lameness (1–3). Lameness/stiffness affects 38% of the zoo elephants, especially males and older individuals (2). Due to the low fertility and high mortality rate of elephant calf, many captive elephant populations decline rapidly (4). Efforts and improvement in medical care, breeding management, and husbandry are being made to increase captive elephant survival.

The gut microbiome is important to host metabolism, immune regulation, and ecological adaptation (5, 6). Gut microbes produce metabolites, neurotransmitters, and bioactive compounds which serve as important regulators (6). Elephants are monogastric herbivorous, hindgut fermenters as horses and rabbits. Their digestive strategy requires them to consume large amounts of low quality forage with only 22% being digested approximately (7). Elephants depend on their intestinal microflora to degrade cellulose due to lack of enzymes (8). Metagenomic sequencing analysis revealed a high diversity of cellulose-degrading bacteria and glycoside hydrolase (GH) family enzymes in Asian elephants (9). Anthropogenic interferences can lead to gut microbiota dysbiosis (5). Overseas translocation, captivity, and deworming all can change the gut microbiota of Asian elephants (5). Health issues common in captive animals may be closely related to the gut microbiome (10). Interventions of gut microbiome may serve as a feasible approach to improve health, survival and reproductive rate of captive elephants (11, 12). In order to develop more effective management strategies to improve the survival of captive elephants, information on their basic biology such as their gut microbiome is needed. Understanding their digestive capabilities could aid in their captive management and conservation. For example, in Asian elephants, the dominant lactic acid bacteria are mainly *Lactobacillales* but not *Bifidobacteriale* (13). When we choose commercial probiotic products to improve the gut health of Asian elephant, this information is important to know beforehand.

Antibiotics are administered for treatment of common syndromes, such as injury, gastrointestinal disease, malnutrition, infectious disease, and ocular disease in elephants (14). For example, Foot problems in captive elephants are considered a significant health issue (2). Antibiotics and anti-inflammatory drugs are normally used to reduce soft tissue swelling and provide analgesia (15). Antibiotic use directly increases antibiotic drug resistance which is mediated by antibiotic resistance genes (16). Furlan et al. (17) reported an extensively drug-resistant *Klebsiella pneumonia* coproducing CTX-M-3, QnrB2, and QnrS1 isolated from an infected elephant (17). Their study highlighted the transmission of extended-spectrum β-lactamases and quinolones resistance producers in captive animals. Zoo animals, especially petting zoo animals, are considered as an emerging reservoir of extended-spectrum β-lactamase and AmpC-producing *Enterobacteriaceae* (18). Ahmed et al. (19) reported that zoo animals are reservoirs of gram-negative bacteria harboring integrons and antimicrobial resistance genes (19). They observed that 21% of the isolates showed resistance phenotypes to two or more antimicrobial agents including ampicillin, cephalothin, streptomycin, trimethoprim-sulfamethoxazole (19). It is necessary to investigate the antibiotic resistance genes profiles of captive elephants.

African and Asian elephant genetically diverged about 7.6 million years ago (20). Although they were in the same captive environment, we hypothesized that zoo African and Asian elephants have distinct profiles of gut microbiome and antibiotic resistance genes. This study aimed to advance our knowledge on gut microbiome and ARGs of captive African and Asian elephants. Results of this study provide a data reference for gut microbiome intervention of captive elephants.

## Materials and methods

### Fecal sample collection

Fecal samples were collected in late October 2021 from three African elephants (*Loxodonta africana*) and four Asian elephants (*Elephas maximus*) in Beijing Zoo. These seven elephants were two male African elephants (FZ-28 years old, relocated from Tanzania to Beijing in 1997; FH-6 years old, born in Beijing zoo), one female African elephant (FJ-28 years old, relocated from Tanzania to Beijing in 1997), one male Asian elephant (YM-21 years old, relocated from Sri Lanka to Beijing in 2007), and three female Asian elephants (YZ-48 years old, relocated from Cambodia to Beijing in 1978; YL-43 years old, relocated from Sri Lanka to Beijing in 1979; YW-20 years old, relocated from Anhui Animal World to Beijing in 2012). Since April all elephants have been on the same diet which included Sudan grass, Leymus chinensis, apples, carrots, watermelon, cucumber, peach, banana, bamboo, and pellet feed. Each elephant was housed in a single pen with a backyard for outdoor activity and a room for rest. The enclosures for African and Asian elephants were far from each other. For African elephants, they could not interact with each other as their pens were not next to each other. For Asian elephants, they can connect each other with their trunk as the backyard was separated with fences.

Fecal samples were collected from the ground before morning within 2 h of defecation. The collected samples were mixed to obtain homogeneity and placed into plastic bags. The samples were transported to the laboratory immediately on ice and stored at −80°C for DNA extraction. All the animals did not receive any antibiotic treatment in the past 6 months before sample collection.

### DNA extraction and Illumina sequencing

Genomic DNA was extracted from the fecal samples with the Magnetic soil and stool DNA Kit (TianGen, China) following the protocols supplied. The purity and integrity of the extracted DNA was examined using 1% agarose gel electrophoresis. The quantity of the extracted DNA was determined using Qubit^®^ dsDNA Assay Kit in Qubit^®^ 2.0 Fluorometer (Life Technologies, CA, USA). Sterile water was used to dilute the samples to obtain an Optical Density (OD) value in the range of 1.8–2.0 for library construction. The library was prepared using the NEBNext^®^ Ultra DNA Library Prep Kit for Illumina (NEB, USA) following the protocol supplied with the kit. Briefly, DNA samples were randomly broken into fragments ~350 bp in length using a Covaris sonicator. The resulting fragments were subjected to end repair, A-tail and sequencing adaptor addition, purification, and PCR amplification for the library construction. Preliminary quantification was obtained using a Qubit^®^ 2.0 Fluorometer (Life Technologies, CA, USA). Then the library was diluted to 2 ng/ul. The insert size of the library was detected using Agilent 2100 and Q-PCR method was used for precise quantification (the effective concentration of the library >3 nM). All libraries were then sequenced on Illumina Hiseq X10 platform with 2 × 150 bp paired reads (Novogene, Beijing, China).

### DNA sequence assembly and annotation

Illumina raw sequencing reads were processed using Readfq (V8) and the obtained clean data was used for subsequent analysis. The specific steps were as follows: (a) remove reads that contain low-quality bases (default quality threshold < 38) exceeding 40 bp; (b) remove the reads with number of N bases higher than 10 bp; (c) remove the reads with 15 bp or more overlap with the adapter. The resulting clean data was assembled using MEGAHIT software (v1.0.4-bata) and then the high-quality reads (99% of raw reads) were assembled into scaftigs (i.e., continuous sequences within scaffolds) (21). Fragments below 500 bp were filtered and the remaining scaftigs were used for subsequent analysis and gene prediction (22). Scaftigs (≥500 bp) from each sample were used to predict ORF (Open Reading Frame) using MetaGeneMark (23). Based on the prediction results, ORFs < 100 nt were discarded (24). To establish a non-redundant gene catalog, the CD-HIT software was used to remove redundancy (clustered at 95% nucleotide identity and 90% coverage) (23, 25, 26), and the longest sequence was selected as the representative sequence. The clean data of each sample was compared to the initial gene catalog using Bowtie2, and the number of reads containing each gene within each sample was calculated. The genes with the number of reads ≤ 2 in each sample were filtered (27). The final gene catalog (UniGenes) was used for subsequent analysis. The abundance of each gene in each sample is calculated based on the number of reads with that gene and gene length aligned.

The UniGenes were compared with sequences from the Non-Redundant Protein Sequence Database (blastp, e value ≤ 1e-5) (22, 28). Sequences having alignment result with e value < 10 times of the minimum e value were retained for subsequent analysis. Based on the annotation results from Least Common Ancestors (LCA) algorithm and gene abundance table, the gene abundance of each sample at each taxonomic level (kingdom, phylum, class, order, family, genus and species) was obtained. The taxa abundance within a sample is the sum of the non-zero number of genes annotated as that taxon (23, 29, 30). The Alpha diversity indices including Shannon, Simpson, Chao1, and Goods-coverage were calculated using the Qiime2 software. The obtained UniGenes were also aligned to the Kyoto Encyclopedia of Genes and Genomes (KEGG) and Carbohydrate-Active EnZymes database (CAZy) functional databases using DIAMOND software (blastp, evalue ≤ 1e-5 (23, 30). Sequences having alignment result with the highest score (one HSP > 60 bits) were used for gene abundance analysis at different functional level in both databases (23, 29, 31–33). The Comprehensive Antibiotic Resistance Database (CARD) contained a great deal of known ARGs and their associated resistant antibiotics (34). The Resistance Gene Identifier (RGI) software was used to align the UniGenes to the CARD database (v2.0.1) and the BLASTP values were set with the parameters of e value < 1e-30 to predict antibiotic resistance genes (35). According to the comparison results of RGI and the abundance information of UniGenes, the relative abundances of each Antibiotic resistance ontology (AROs) was calculated to obtain the abundance of ARGs.

### Statistical analysis

For statistical analysis, the data was not transformed or normalized except during the calculation of Bray-Curtis distance. Venn diagrams of UniGenes and ARGs from both host species were drawn with the VennDiagram package in R (v3.4.1). Alpha diversity data were analyzed using the PROC GLIMMIX procedure of SAS (SAS Institute, Inc., Cary, NC, USA) including host species as fixed effect in the model. To reveal the gene abundance correlation between samples, we calculated the pairwise Spearman's rank correlation and generated the correlation coefficient heatmap (pheatmap package, R 3.4.1). MetaStats analysis was used to look for different taxa at the phylum level, family level and genus levels and different functions in level 1 and 2 of KEGG and CAZy databases. Permutation test between groups was used in the MetaStats analysis to get the *P*-value. The Story and Tibshirani algorithm was used to correct the *P*-value and acquire q value, also known as False Discovery Rate (36–38). For microbial composition and functional profiles between African and Asian elephants, we also performed principal coordinates analysis (PCoA) based on Bray-Curtis distance (ade4 and ggplot2 package, R v3.4.1). Then permutational multivariate ANOVA (PERMANOVA, 999 permutations) on Bray-Curtis dissimilarity matrices was conducted to investigate the dissimilarity of microbial composition and functional profiles between the host species (Bray-Curtis distance, permutation # = 999, vegan package, R 3.4.1) (39). LEfSe analysis of different taxa was conducted using the LEfSe software (default LDA score was set at 4) (40). The microbial composition between groups was also tested using the similarity analysis with the “anosim” function (R vegan package, 3.4.1). The Circos software was used to construct the Circos diagram to reveal the relationship of different resistance mechanism and the corresponding taxa (41).

## Results

### Metagenome sequencing and assembly

DNA samples recovered from seven elephants' fecal samples were subjected to high throughput sequencing and total of 44.49 G data were obtained. 44.45 Gbase of clean data was obtained after low quality data being filtered. The average total length, average length, and N50 length were 363,007,716, 1,408, and 1,792 bp, respectively. The average number of scaftigs for all samples was 260,068. The gene prediction and abundance analysis results showed total number of non-redundant genes was 1,900,387 and the total and average length of genes were 1,265.69 Mbp and 666.01 bp. The average gene numbers for African and Asian elephants were 768,051 and 922,391, respectively. Predicted genes were annotated in three databases, about 52.88% in the KEGG database, 3.68% in the CAZy database and 0.025% in the CARD database.

**Figure 1:**
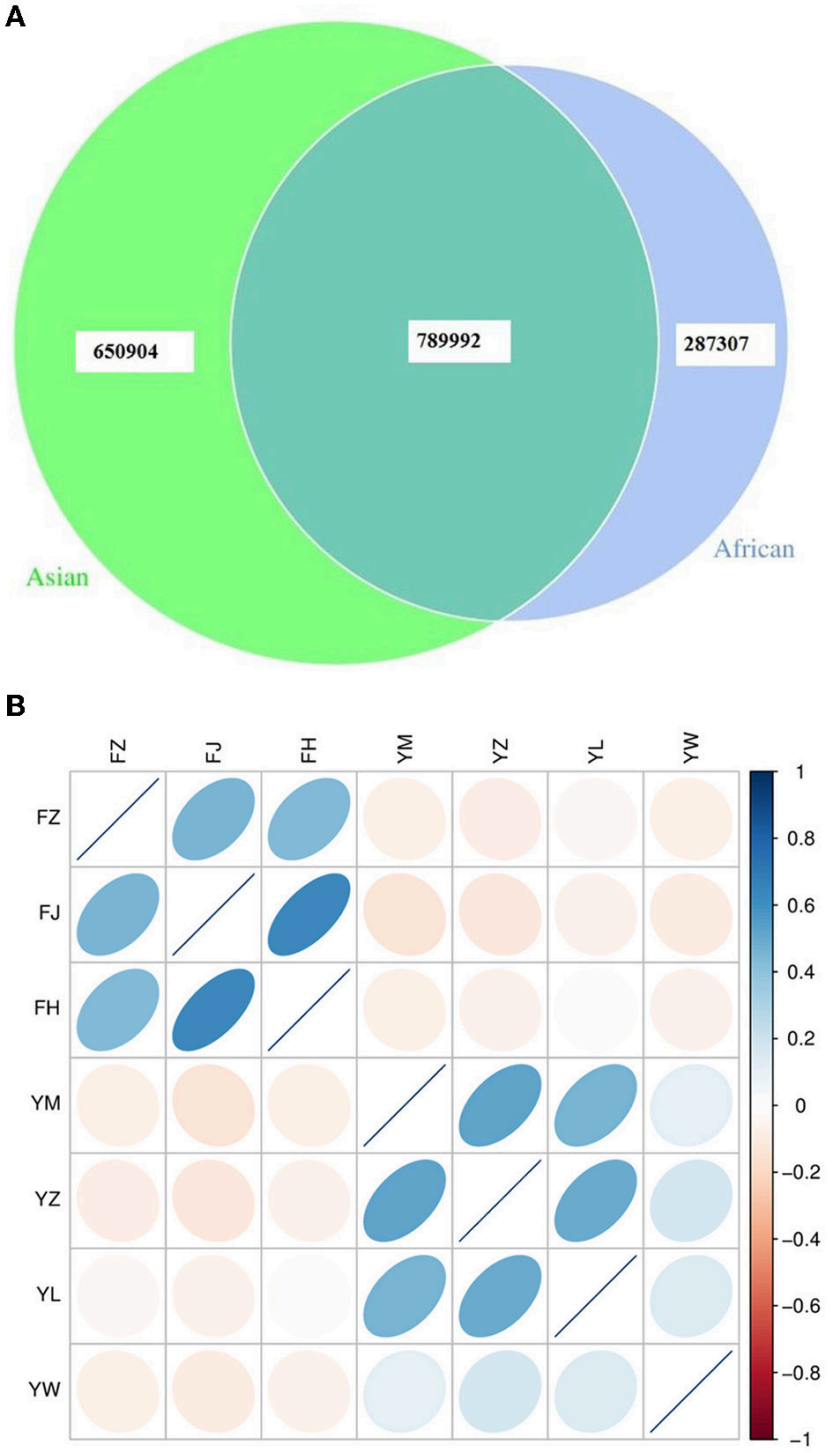
**(A)** Venn diagram of gene numbers between African and Asian elephants; **(B)** Correlation heatmap of gene abundance between samples from African and Asian elephants. The color legend on the right shows the level of correlation coefficient. The ellipse with the darker color means the higher absolute value of the correlation coefficient between samples. The right deviation of the ellipse indicates that the correlation coefficient is positive, and the left deviation is negative. The flatter the ellipse, the greater the absolute value of the correlation coefficient. FZ, FJ, and FH are African elephants. YM, YZ, YL, and YW are Asian elephants.

African and Asian elephants shared 45.7% of total genes annotated (789,992 genes shared, Figure 1A). The numbers of specific genes in African and Asian elephants were 287,307 and 650,904 respectively. Samples from the same host species were positively correlated and sample from different host species were negatively correlated (Figure 1B). Among the three African elephants, samples from FH and FJ had the highest correlation coefficient (*r* = 0.64) as FJ was the mother of FH. Interestingly, samples from FH and FZ only had a correlation coefficient of 0.43 as FZ was the father of FH. Samples from FZ and FJ had a correlation coefficient of 0.46. Compared to African elephants, samples from the four Asian elephants had lower correlation coefficients from 0.11 to 0.52. These four Asian elephants were not genetically related to each other. The absolute values of correlation coefficients between African and Asian elephants were all below 0.14.

### Gut microbial communities of African and Asian elephants

**Figure 2:**
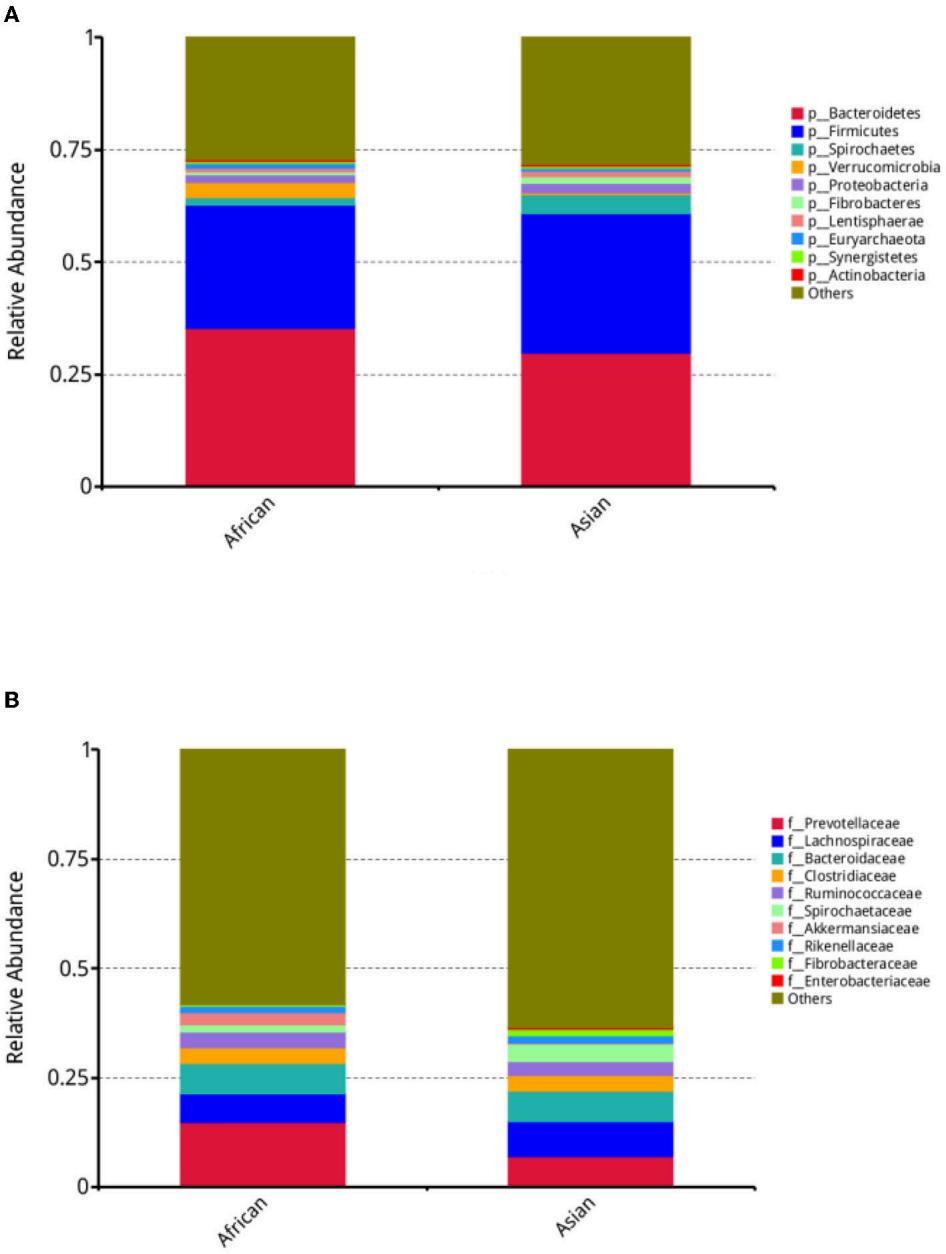
**(A)** Stacked bar plot depicting relative abundance of top ten bacteria phyla; **(B)** Stacked bar plot depicting relative abundance of top ten bacteria families in African (*n* = 3) and Asian (*n* = 4) elephants.

In our study, about 11,132 taxa were identified in both African and Asian elephants and there were about 58.2% and 59.9% of the taxa unidentified in the two host species, respectively. The average numbers of assembled scaftigs for African and Asian elephants were 224,249 and 286,932 respectively. Alpha diversity, including Shannon, Simpson, Chao1 and Goods coverage indices were not different between the two groups (Supplementary Table 1). For bacteria at the phylum, family, and genus levels, MetaStats analysis results on the top ten taxa were presented (Supplementary Table 1). At the phylum level, the relative abundance of *Spirochaetes* in African elephants was significantly lower than Asian elephants (1.69 vs. 4.22%, *FDR* = 0.00, Figure 2A, Supplementary Table 1). African elephants had significantly higher relative abundance of *Verrucomicrobia* than Asian elephants (3.33 vs. 0.50%, *FDR* = 0.01; Figure 2A, Supplementary Table 1). At the family level, the relative abundance of *Spirochaetaceae* in African elephants was significantly lower than Asian elephants (1.63 vs. 4.11%, *FDR* = 0.01; Figure 2B, Supplementary Table 1). The relative abundance of *Akkermansiaceae* in African elephants was significantly higher than that in Asian elephants (2.90 vs. 0.07%, *FDR* = 0.02; Figure 2B, Supplementary Table 1). At the genus level, the relative abundance of *Treponema* and *Akkermansia* in African elephants were significantly lower and higher than those in Asian elephants, respectively (1.56 vs. 3.98%, *FDR* = 0.02; 2.89 vs. 0.07%, *FDR* = 0.02, Supplementary Table 1).

**Figure 3:**
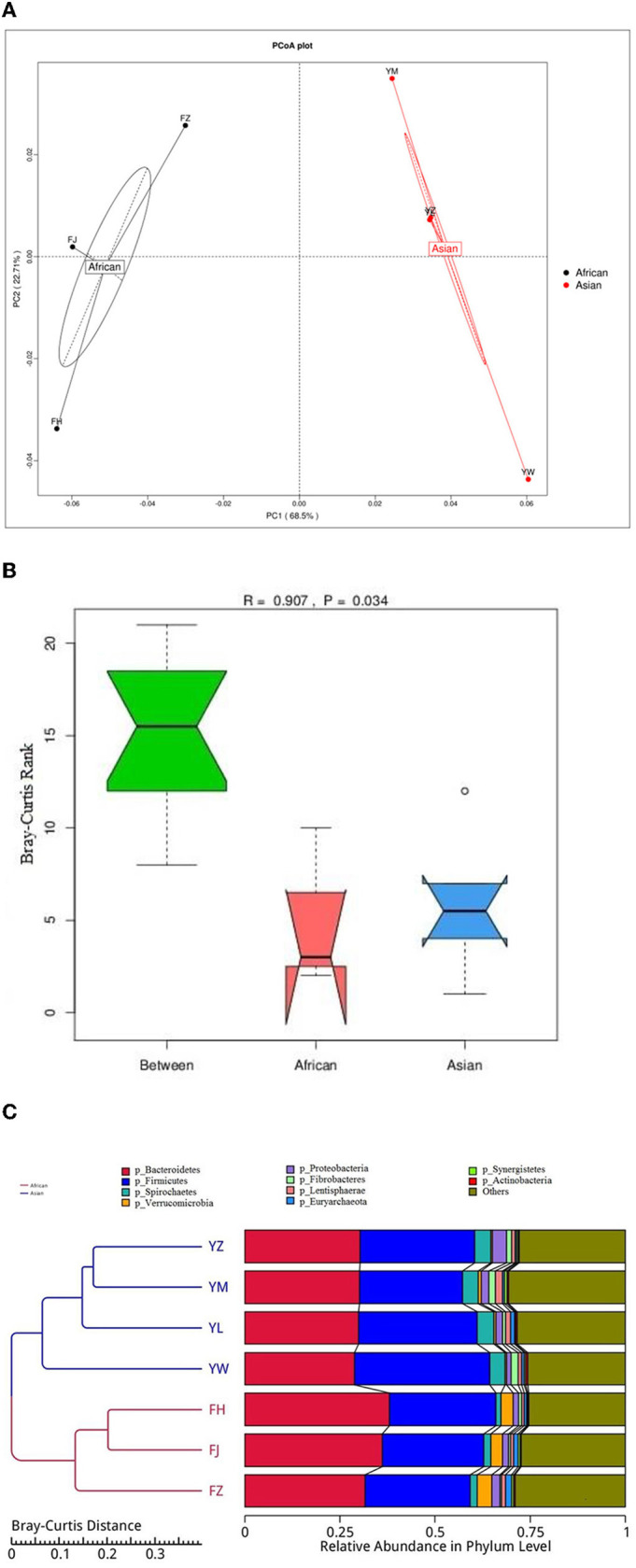
**(A)** Principal Co-ordinates Analysis **(**PCoA) of microbiota from African and Asian elephants at the phylum level (Bray-Curtis distance); **(B)** ANOSIM analysis of microbial composition at the phylum level. The y-axis of the plot represents the ranked Bray-Curtis distance. Between is the combined microbial information at the phylum level from both groups. A higher median line of between than the median line of the other two groups means that the variance between groups was higher than variance within groups; **(C)** Cluster tree based on Bray-Curtis distance at the phylum level. The left side is the Bray-Curtis distance clustering tree structure; the right side is the relative abundance distribution map of each sample at the phylum level.

**Figure 4:**
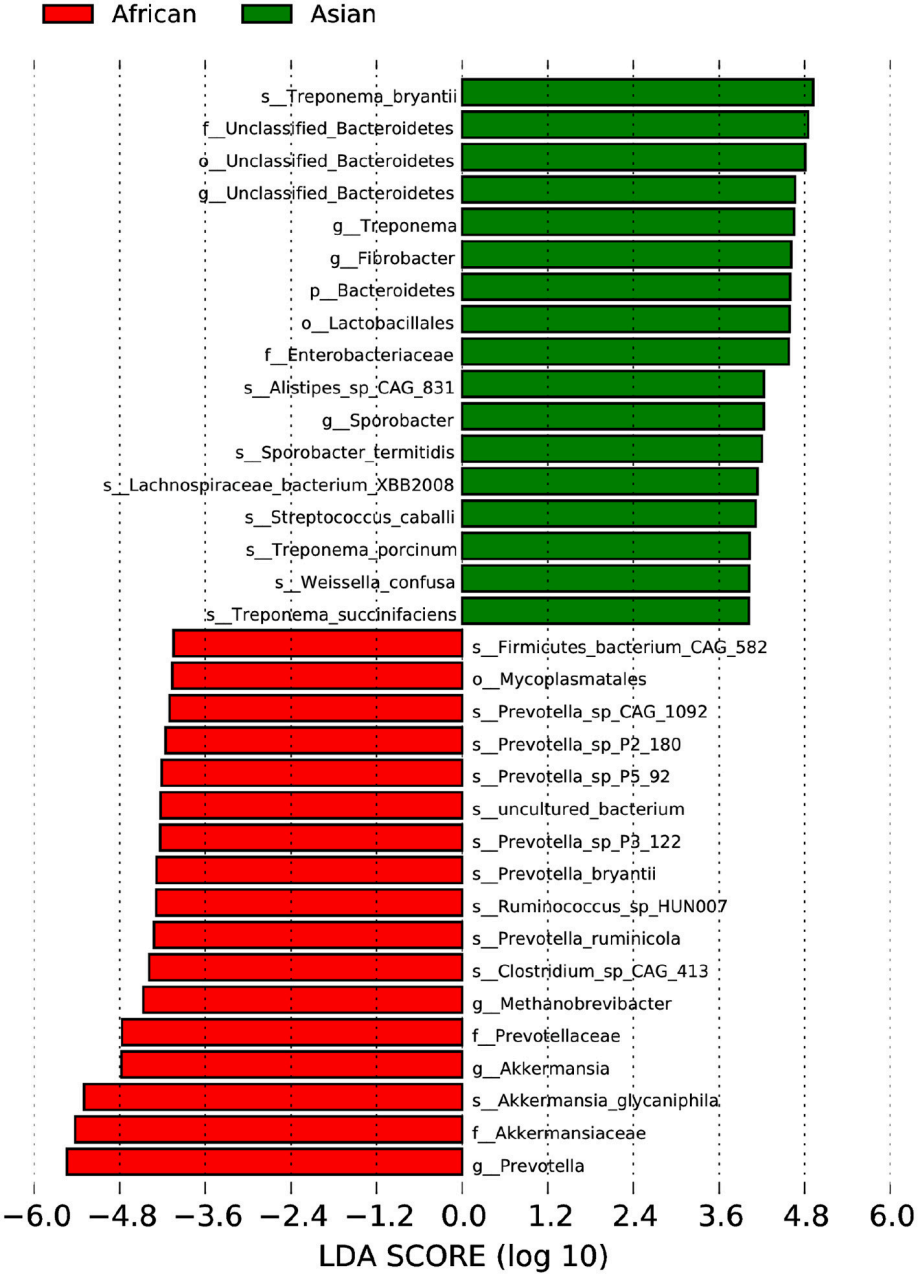
LEfSe Analysis of Differential taxa between Groups is shown in the LDA value distribution histogram. The presented are biomarkers taxa enriched within each group (LDA > 4). The length of the histogram (LDA score) represents the effect size of each abundant taxa.

African and Asian elephants had distinct gut microbial composition according to the results of principal component analysis (PCoA) as samples from the two groups were clearly separated (Figure 3A). Moreover, the PERMANOVA analysis showed that host species (R^2^ = 0.63, *P* = 0.03) was a strong predictor influencing microbial community structures. The first and second principal components explained 68.5 and 22.7% of the variation in the samples. Regarding the ANOSIM analysis at the phylum level, the *R*-value was >0 indicating the between group variation was higher than within group variation (*R* = 0.91). A *P*-value of 0.03 indicated a significant difference between African and Asian elephants in the microbial communities (Figure 3B). The cluster tree based on Bray-Curtis distance at the phylum level also showed that samples were categorized by host species (Figure 3C). The LDA value distribution histogram showed the enriched biomarker species in African and Asian elephants (Figure 4). The enriched species within each host species were mainly at the genus and species level.

### Metabolism and function of the gut microbiome of African and Asian elephants

**Figure 5:**
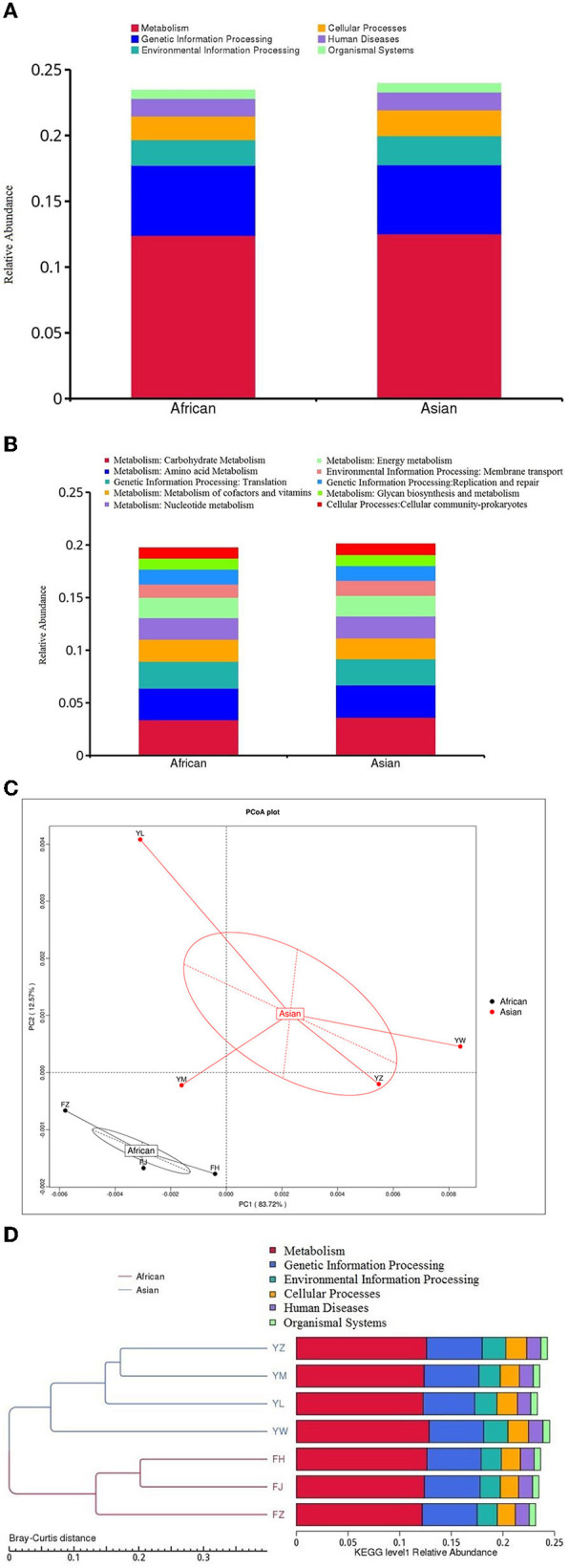
**(A)** Relative gene abundance bar plot of functional annotations at level1in KEGG database; **(B)** Relative gene abundance histogram of top ten functional annotations at level 2 in KEGG database in African (*n* = 3) and Asian (*n* = 4) elephants. **(C)** PCoA analysis of functional gene abundance at level 1 in KEGG database based on Bray-Curtis distance **(D)** Clustering tree based on Bray-Curtis distance (the left side is the clustering tree structure; the right side is the functional relative abundance distribution of each sample at the first level of KEGG).

Among the top ten functional subcategories at level 2 of KEGG database, MetaStats analysis showed that relative gene abundance of cellular community-prokaryotes, membrane transport, and carbohydrate metabolism in African elephants were significantly lower than that in Asian elephants (0.98 vs. 1.03%, *FDR* = 0.04; 1.25 vs. 1.43%, *FDR* = 0.03; 3.39 vs. 3.63%, *FDR* = 0.02; Figure 5B, Supplementary Table 1). Although it was statistically significantly different, it should be noted that the actual difference between the two groups was small. None of the six functional categories at level 1 of KEGG database was different between the two host species (Figure 5A). PCoA analysis of gene abundance at level 1 of KEGG database showed that the samples from African elephants and Asian elephants were clearly separated (Figure 5C). The first and second principal components explained 83.72 and 12.57% of the variation, respectively. The PERMANOVA analysis showed that host species (*R*^2^ = 0.32, *P* = 0.20) did not significantly affect the microbial functions at level 1 of KEGG database. The clustering tree based on Bray-Curtis distance showed that samples from African and Asian elephants formed two clusters (Figure 5D).

**Figure 6:**
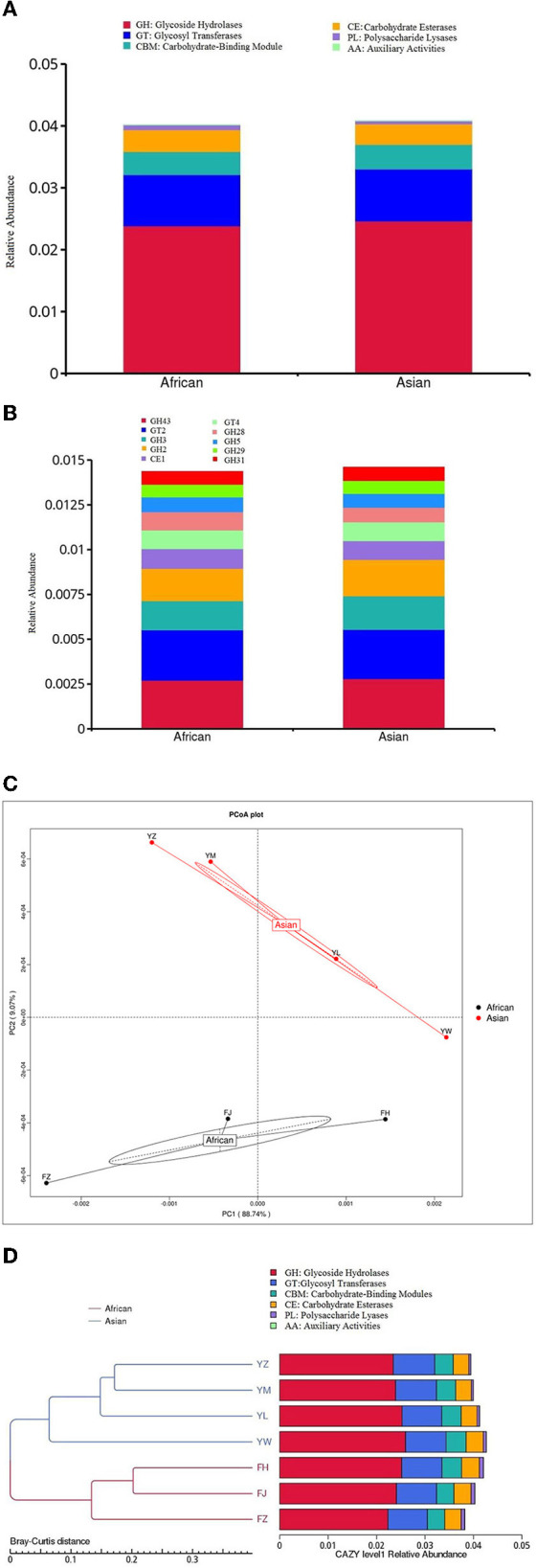
**(A)** Relative gene abundance bar plot of functional annotations at level 1 in CAZy database; **(B)** Relative gene abundance histogram of top ten functional annotations at level 2 in CAZy database in African (*n* = 3) and Asian (*n* = 4) elephants. **(C)** PCoA analysis of functional gene abundance at level 1 in CAZy database based on Bray-Curtis distance. **(D)** Clustering tree based on Bray-Curtis distance (the left side is the clustering tree structure; the right side is the functional relative abundance distribution of each sample at the first level of CAZy).

None of the six functional categories at level 1 of CAZy database was significantly different between the two host species (Figure 6A). Among the top ten functional subcategories at level 2 of CAZy database, MetaStats analysis showed that African elephants samples had higher relative gene abundance of GH 28 compared to Asian elephants (0.10 vs. 0.08%, *FDR* = 0.03; Figure 6B, Supplementary Table 1). PCoA analysis of gene abundance at level 1 of CAZy database based on Bray-Curtis distance showed that samples from African elephants and Asian elephants can be clearly separated (Figure 6C). The first and second principal components explained 88.74 and 9.07% of the variation, respectively. The PERMANOVA analysis showed that microbial functions at level 1 of CAZy database were not significantly different between the two host species (*R*^2^ = 0.13, *P* = 0.47). The clustering tree based on Bray-Curtis distance showed that samples from African and Asian elephants formed two clusters (Figure 6D).

### Antibiotic resistance genes (ARGs) profile of African and Asian elephants

**Figure 7:**
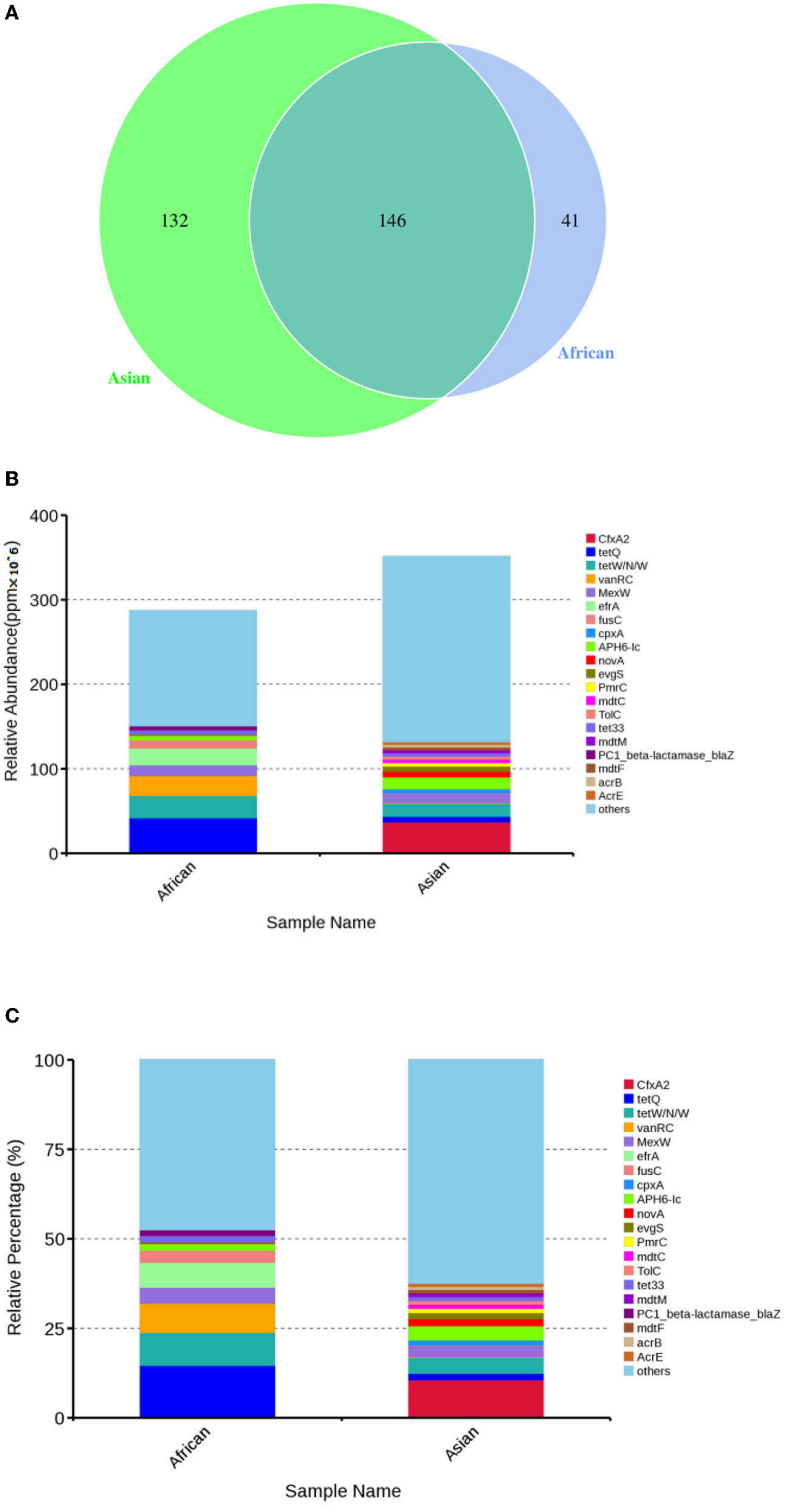
**(A)** Venn diagrams of antibiotic resistant genes shared by African and Asian elephants and specific genes belong to each host species; **(B)** Absolute gene abundance of different ARGs in each sample (× 10^6^); **(C)** Relative abundance of top 20 ARGs.

**Figure 8:**
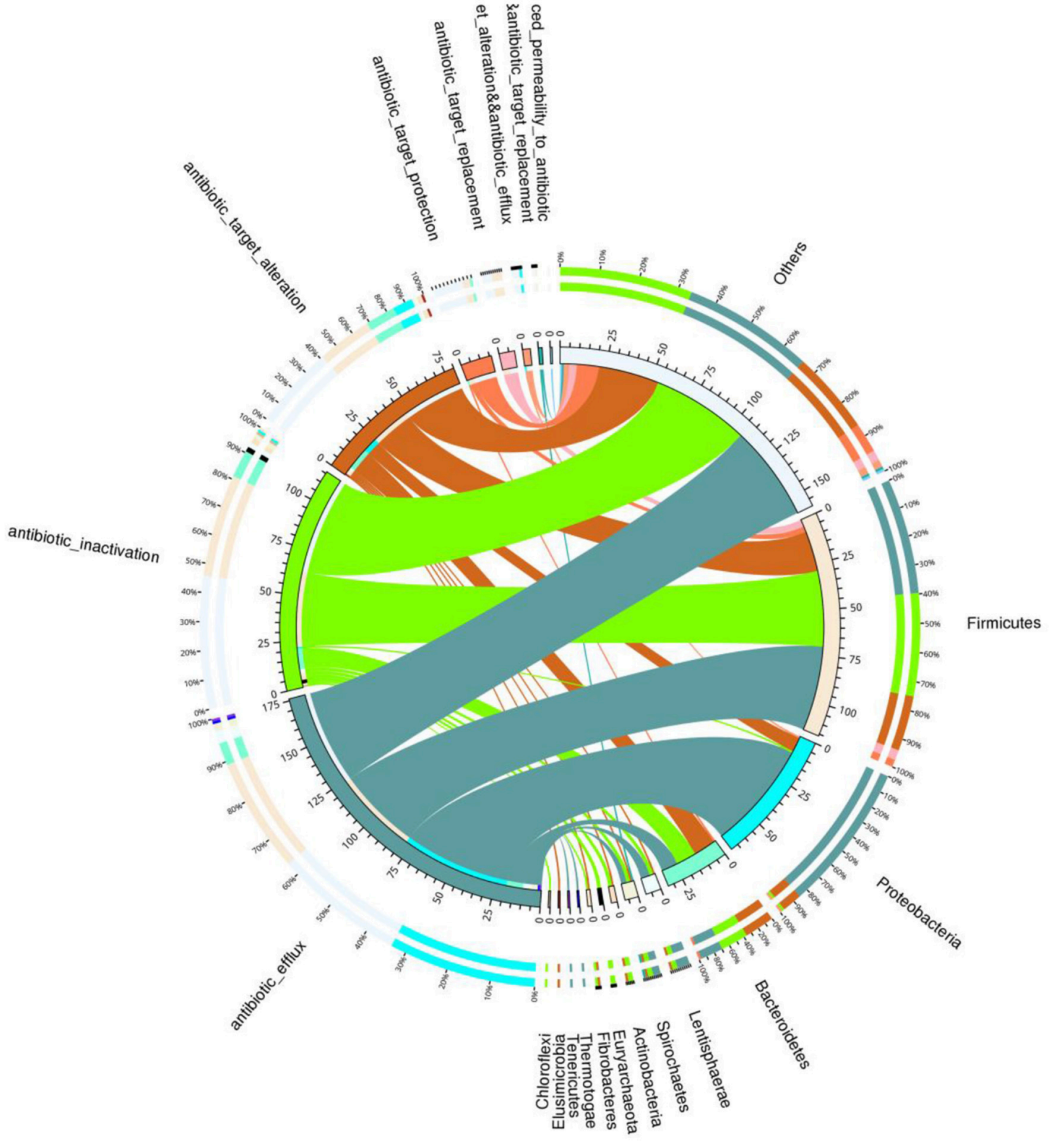
Circos diagram summarizing the relationship between the resistance mechanism and the microbial taxa (combined data from both African and Asian elephants). The circos diagram is divided into two parts, with phylum-level taxa information on the right and resistance mechanism information on the left. For the outer circle, the left side is the relative proportion of the resistance genes coding for corresponding resistance mechanism within each taxa. The right side is the relative proportion of the resistance genes coding each resistance mechanism within the taxa. For the inner circle, different colors represent different taxa and resistance mechanisms, and the scale is the number of genes. The left side is the sum of the number of resistance genes coding corresponding resistance mechanism in different taxa. The right side is the sum of the number of resistance genes coding different resistance mechanisms within each taxa.

Total of 146 ARGs (45.76% of total ARGs), annotated against CARD database, were shared by African and Asian elephants. Other than the 146 ARGs shared, 132 specific ARGs were observed in samples from Asian elephants and 41 in samples from African elephants (Figure 7A). MetaStats analysis showed that samples from African elephants had significantly higher relative abundance of *vanO* (6.68 × 10^−6^ ppm vs. 1.23 × 10^−6^ ppm, *FDR* = 0.00), *tetQ* (4.08 × 10^−5^ ppm vs. 6.30 × 10^−6^ ppm, *FDR* = 0.04), and *efrA* (2.00 × 10^−5^ ppm vs. 0 ppm, *FDR* = 0.04) than Asian elephants (Supplementary Table 1). It should be noted that the relative abundance of ARGs from Asian elephants were higher than that from African elephants (Figure 7B). Based on the circos diagram (Figure 8), the top five antibiotic resistance mechanisms were antibiotic efflux, antibiotic inactivation, antibiotic target alteration, antibiotic target protection, and antibiotic target replacement. ARGs encoding for antibiotic target alteration were mainly from *Firmicutes* and others following by *Proteobacteria*, and *Bacteroidetes* (Figure 8). Antibiotic resistance genes coding for antibiotic efflux were mainly from *Firmicutes, Proteobacteria*, and others following by *Bacteroidetes*. *Firmicutes* and others also had higher proportions of ARGs with the antibiotic inactivation mechanism, followed at a distance by *Bacteroidetes*.

## Discussion

### Gut microbial communities of African and Asian elephants

Many factors affect the gut microbiome including diet, host genetics, and environment (42, 43). Diet affects the diversity and composition to a larger extent than other factors as different food substrates promote growth of different microbial taxa, leading to taxonomic variation (44). Host species influence the microbiota at finer scales of taxonomic ranks such as family, genera, ASV etc., (45, 46). Through genes associated with the immune system, host phylogeny also can influence the microbiome (47). In our study, African and Asian elephants had different gene abundance patterns. This also has been shown previously by Keady et al. (12) that African and Asian elephants have different microbial structure and dissimilar bacterial composition (12). The difference was mainly caused by the host species as the PERMANOVA analysis showed that the host species explained 63% of the variation. However, caution is needed in interpreting the results as PERMANOVA test behave unreliably for unbalanced designs in the face of heterogeneity (48) and this was our case due to the small sample size in each group.

Both Asian elephant and African elephant originated in Africa. Due to the habitat and dietary difference between Asian and African elephants, they have evolved to the two species in African and Asia. In wild, the Asian elephant's diet is made up of a greater proportion of grass and the African's of leaves (49). African elephants tend to have more stable, hierarchal, and close knit social groups (50). Both factors may contribute to the difference of gut microbial community during evolution. Four bacterial phyla are core members of the mammalian gut microbiome including *Bacteroidetes, Firmicutes, Proteobacteria* and *Actinobacteria* (51). Consistent with previous research findings (5, 12, 52, 53), our results showed the gut microbiome of captive African and Asian elephants was dominated by two phyla: *Bacteroidetes* and *Firmicutes*, following by *Spirochaetes, Verrucomicrobia* and *Protrobacteria* (Figures 2A, B). Compared to dominant phyla in other animals like cow (*Clostridiales* and *Prevotellaceae*) or termite (*Spirochetes* and *Fibrobacteres*), the dominant phyla in elephants allow them digest various plant biopolymers (9, 54). Jakeer et al. (55) reported that *Proteobacteria* was the most abundant phylum (91%), followed by *Actinobacteria* (4.6%) and *Bacteroidetes* (2.5%) which was very different from our results (55). In that study, the only Asian elephant sampled was on green fodder, tree fodder, dry fodder, banana, jaggery, mung bean, rice, pearl millet, turmeric, salt, and mustard oil for 3 months before sample collection. This difference can be caused by diet difference or low sample size.

Captivity can change the composition of gut microbiota (56). In captive environment, their diet is less diverse compared to that in wild environments. In our study, the captivity time for each elephant was different. Two of the African elephants were translocated to Beijing zoo in 1997 from Tanzania and one African elephant was born in the zoo. The four Asian elephants were translocated to Beijing zoo from different countries (Sri Lanka, and Cambodia, China) at different times (2007, 1979, 1978, and 2007). Furthermore, these elephants were at different ages and we were not aware of their disease and treatment histories. All these factors could contribute to the difference of their gut microbiome.

### Metabolism and function of the gut microbiome of African and Asian elephants

Elephants are the largest land herbivorous animals and they derive energy from plant-based diet. Elephants do not secrete enzymes to break down plant cell walls and rely on anaerobic microbe to ferment the consumed forage into a digestible form (8, 52). More important to the baby elephants, gut microbes from either its mother or other members' feces are necessary for digestion of plant matter (9). In the wild, diet of Asian elephants consists of tree barks, stems, roots, leaves and shrubs (57). Asian elephants in Xishuangbanna wild elephant valley feed on mostly bamboo and wild plantains (52). Thus elephants have evolved to encode unique CAZymes to hydrolyze plant-derived polymers fast and efficiently (55). Carbohydrate active enzymes (CAZymes) include glycosyltransferases (GTs), glycoside hydrolases (GHs), carbohydrate-binding modules (CBMs), polysaccharide lyases (PLs), and carbohydrate esterases (CEs) (58). Jakeer et al. (55) suggested that adult elephant gut microbiome could be a potential source of CAZymes for *in vitro* biomass hydrolysis (55). Bacteroidales had a high coverage of GH 5 and GH 9 genes making it the main cellulose degraders in the elephant gut (9). Our results showed that glycoside hydrolases (GH) was the most abundant and glycosyltransferases (GT) was the second most abundant of CAZymes in both African and Asian elephants. This is different from Jakeer et al. (55)'s findings whereas GT was the most abundant enyzmes (34%) (55). We observed that only GH 28 was significantly different in African and Asian elephants. GH 28 is a set of structurally related glycoside hydrolases enzymes that hydrolyze glycosidic bonds in pectin (59). This functional difference may indicate a different in energy allocation and metabolic capabilities between African and Asian elephants. Based on the PERMANOVA analysis, host species did not significantly affect the microbial functions as it only explained 32% (level 1 of KEGG, *P* = 0.20) and 13% (level 1 of CAZy, *P* = 0.47) of the functional variation. The clear visual separation between groups in the PCoA plots (Figures 5C, 6C) contradicts with the PERMANOA results. PCoA is a method for dimension reduction and the plots were looking at the top two axes, while separation may be different on a different axis. The low sample size could be another reason for this contradiction. In addition, the *P*-values of PERMANOVA (>0.05) might explained why the gene difference of cellular community- prokaryotes, membrane transport, carbohydrate metabolism, and GH 28 were small between the two species although they were shown significantly different by MetaStats analysis.

### Antibiotic resistance genes (ARGs) profile of African and Asian elephants

The emergence of antibiotic resistance is a growing threat to public health worldwide and other animals (60). Compared to the gram positive bacteria, gram negative bacteria acquire resistance faster thus multidrug resistant negative bacteria pose the biggest threat to public health (61). Wild animals act as efficient antimicrobial resistance reservoirs and epidemiological links between human, livestock and the environment (62–64). Drug resistance in wildlife can develop on its own or by exposure to human waste or agricultural runoff with antibiotic residues (62). Compared to wild animals, zoo animal populations are more closely associated with human populations, thus it is highly likely that antimicrobial resistance (AMR) and ARGs in zoo animals are more similar to humans and livestock. Limited studies have been conducted to monitor antibiotic resistant bacteria in zoo animals.

Based on the medical records from the zoo keeper, all seven elephants did not receive antibiotic treatment for 6 months before sample collection. The four Asian elephants received antibiotic treatment in February and March which was about seven months prior to sample collection. Some antibiotic resistance genes do not vanish immediately after antibiotics end as some bacteria can pass on those genes to the next generation (65). We expected samples from Asian elephants might have higher numbers of ARGs. And this was indeed observed that the total relative abundance (ppm) of Asian elephants were higher than that of African elephants (Figures 7A, B). The fact that samples from African elephants had significantly higher abundance of *vanO, tetQ*, and *erfA* is intriguing. Furthermore, animal food may also play a role in disseminating antibiotic resistance to zoo animals as antibiotic resistant bacteria and ARGs have been isolated from zoo animal foods (66). Heavy metals can drive the co-selection of antibiotic resistance in soil and water bodies (67). As African elephants ate more food than Asian elephants, the higher intake of heavy metals from food might be causing higher abundance of *vanO, tetQ*, and *erfA* and this needs further investigation.

## Conclusions

In conclusion, it was observed that the captive African and Asian elephants on the same diet have distinct gut microbial communities. The observed higher ARG abundance of *vanO, tetQ*, and *erfA* in African elephants needs more investigation. Other than the host species effects on gut microbiota, influences of other putative factors should also be considered. Our findings established the ground work for future research on improving gut health of captive elephants.

## Data availability statement

The data that support the findings of this study can be found at http://www.ncbi.nlm.nih.gov/bioproject/930555. Reference number (PRJNA930555).

## Ethics statement

The animal study was reviewed and approved by Institutional Animal Care and Use Committee of Foshan University (Foshan, China).

## Author contributions

XF and RH drafted the manuscript. TL and YL conducted the study and collected samples. CL, XC, and WZ analyzed the samples. YW and HZ performed data analysis. YL reviewed and revised the manuscript. All authors read and approved the final manuscript.
